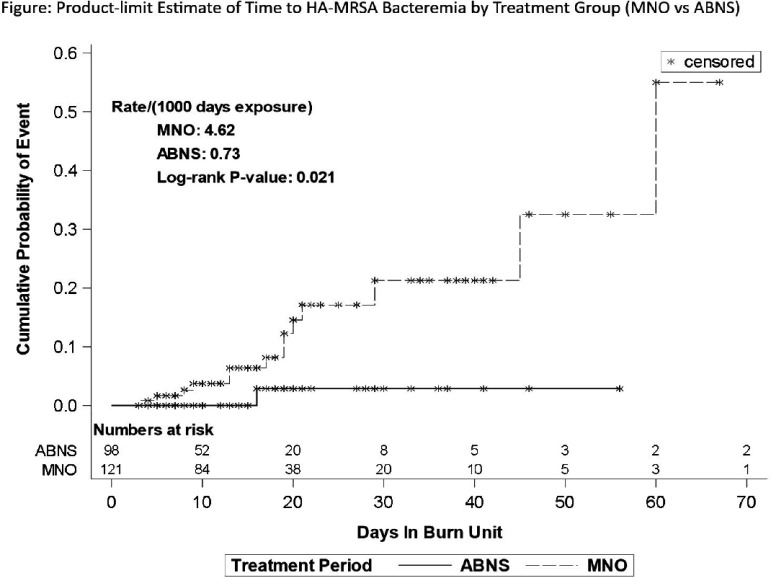# Alcohol-Based Nasal Sanitizer Provides Superior Protection Against MRSA Bacteremia Compared to Mupirocin in Burn Patients

**DOI:** 10.1017/ash.2025.192

**Published:** 2025-09-24

**Authors:** Werner Bischoff, Tamika Lovelace, Timothy Craven, Xiaogang Wu

**Affiliations:** 1 Wake Forest University School of Medicine; 2 infection prevention; 3 Department of Biostatistics and Data Science; 4 Atrium Health Wake Forest Baptist

## Abstract

**Background:** Burn injuries pose a significant risk for infections. Nasal decolonization with mupirocin nasal ointment (MNO) is an established method to prevent infections with Methicillin-resistant Staphylococcus aureus (MRSA). We compared the effectiveness of an alcohol-based nasal sanitizer (ABNS) to MNO against MRSA bacteremia in burn patients. **Methods:** This was a retrospective before/after study comparing the impact of an MNO (study arm 1; Bactroban 2%, GlaxoSmithKline, NC; application: twice daily for five days after admission) and an ABNS (study arm 2; Nozin, Bethesda, MD; application: twice daily for entire stay on Burn unit) on Healthcare Associated (HA-) MRSA bacteremia events in burn patients. The Burn unit consists of eight intensive care beds for burn care and 15 regular beds in an 885 bed, tertiary care, academic hospital. Inclusion criteria were all burn patients 18 years of age and older admitted under the burn service for more than four consecutive days. No mandatory MRSA screening was performed. Outcome measure was HA-MRSA bacteremia acquired > four days after admission. Patient characteristics included demographics, BMI, intensive care need, MRSA colonization at admission, type and degree of burn, inhalation injury, total burn surface area, Baux score, inpatient mortality, length of stay by total, burn mixed acuity and burn ICU were documented. Daily compliance with treatments was extracted from patient records (EPIC, Verona, WI). Continuous patient characteristics were compared using t-tests or Wilcoxon signed-rank test (for factors with skewed distributions), and chi-square tests for categorical factors. Product-limit time-to-event analysis and log-rank test were used to compare the outcome measure between groups. **Results:** From 08/01/2021 to 07/31/2024 a total of 920 patients were enrolled (MNO arm: 448; ABNS arm: 462) with 239 and 217 meeting inclusion criteria. No differences in patient characteristics were detected between the two groups at all patients and >80% treatment compliance levels (MNO: 121 encounters; ABNS: 98 encounters). Patients in the MNO arm encountered 14 events compared to one event in the ABNS arm (p=0.0021). The figure displays the product-limit time to event estimates for developing HA-MRSA bacteremia at the >80% adherence level (p= 0.021). Lower adherence levels (50%, 60%, 70%) did not show significance (p>0.05) in the time-to-event analysis. **Conclusion:** Providing ABNS >80% of the time resulted in a significant decrease in HA-MRSA bacteremia events in burn patients compared to an MNO. The daily application throughout hospitalization may offer additional protection against MRSA in patients hospitalized for extended periods of time.

**Figure:**